# Identifying the Causes of Unexplained Dyspnea at High Altitude Using Normobaric Hypoxia with Echocardiography

**DOI:** 10.3390/jimaging10020038

**Published:** 2024-01-31

**Authors:** Jan Stepanek, Juan M. Farina, Ahmed K. Mahmoud, Chieh-Ju Chao, Said Alsidawi, Chadi Ayoub, Timothy Barry, Milagros Pereyra, Isabel G. Scalia, Mohammed Tiseer Abbas, Rachel E. Wraith, Lisa S. Brown, Michael S. Radavich, Pamela J. Curtisi, Patricia C. Hartzendorf, Elizabeth M. Lasota, Kyley N. Umetsu, Jill M. Peterson, Kristin E. Karlson, Karen Breznak, David F. Fortuin, Steven J. Lester, Reza Arsanjani

**Affiliations:** 1 Aerospace Medicine Program, Department of Internal Medicine, Mayo Clinic, Scottsdale, AZ 85054, USA; 2 Department of Cardiovascular Medicine, Mayo Clinic, Scottsdale, AZ 85054, USA; 3 Department of Cardiovascular Medicine, Mayo Clinic, Rochester, MN 55905, USA

**Keywords:** echocardiography, stress test, high altitude, dyspnea, hypoxic simulation testing

## Abstract

Exposure to high altitude results in hypobaric hypoxia, leading to physiological changes in the cardiovascular system that may result in limiting symptoms, including dyspnea, fatigue, and exercise intolerance. However, it is still unclear why some patients are more susceptible to high-altitude symptoms than others. Hypoxic simulation testing (HST) simulates changes in physiology that occur at a specific altitude by asking the patients to breathe a mixture of gases with decreased oxygen content. This study aimed to determine whether the use of transthoracic echocardiography (TTE) during HST can detect the rise in right-sided pressures and the impact of hypoxia on right ventricle (RV) hemodynamics and right to left shunts, thus revealing the underlying causes of high-altitude signs and symptoms. A retrospective study was performed including consecutive patients with unexplained dyspnea at high altitude. HSTs were performed by administrating reduced FiO_2_ to simulate altitude levels specific to patients’ history. Echocardiography images were obtained at baseline and during hypoxia. The study included 27 patients, with a mean age of 65 years, 14 patients (51.9%) were female. RV systolic pressure increased at peak hypoxia, while RV systolic function declined as shown by a significant decrease in the tricuspid annular plane systolic excursion (TAPSE), the maximum velocity achieved by the lateral tricuspid annulus during systole (S’ wave), and the RV free wall longitudinal strain. Additionally, right-to-left shunt was present in 19 (70.4%) patients as identified by bubble contrast injections. Among these, the severity of the shunt increased at peak hypoxia in eight cases (42.1%), and the shunt was only evident during hypoxia in seven patients (36.8%). In conclusion, the use of TTE during HST provides valuable information by revealing the presence of symptomatic, sustained shunts and confirming the decline in RV hemodynamics, thus potentially explaining dyspnea at high altitude. Further studies are needed to establish the optimal clinical role of this physiologic method.

## 1. Introduction

Mountainous areas are a frequent destination for travel, seasonal work, and residence. Globally, more than 100 million people visit high-altitude regions (≥2500 m, approximately 8000 ft) every year, and it is estimated that 140 million people have a permanent high-altitude residence [1,2]. Furthermore, the yearly number of passengers carried on scheduled flights worldwide is 4.5 billion, and aircrafts cabin pressurization (contingent on flight altitude and aircraft) is equivalent to an altitude of 5000–8000 ft during flight [3,4]. At these elevations, the partial pressure of oxygen (O_2_) in the atmosphere falls along with the barometric pressure [5], leading to a reduction in alveolar partial O_2_ pressure (PAO_2_) compared to sea level. Consequently, the acute decline in arterial pressure of O_2_ (PaO_2_) initiates a series of physiological responses to maintain an adequate O_2_ delivery to the tissues [6], including primarily hyperventilation, increase in heart rate, and pulmonary vasoconstriction [7]. Hypoxic pulmonary vasoconstriction is, in effect, driven by the fall in PAO_2_ pressure and can affect the entire pulmonary vascular bed [7,8]. One critical goal of the hypoxic vasoconstriction is to preserve the local ventilation-perfusion matching and enhance blood O_2_ levels. However, in the setting of hypoxia during high-altitude exposure, it can result in an exaggerated increase in pulmonary vascular resistance (PVR), leading to higher right ventricular (RV) pressure [9].

A subset of the population suffers from limiting symptoms when rapidly ascending to high elevations, including dyspnea, fatigue, and exercise intolerance [10]. These individuals are often characterized by an exaggerated arterial hypoxemia at high altitude, which can initiate a maladaptive response [11]. The underlying mechanisms for this maladaptive response are incompletely understood [12], and it is still under debate why some patients are more susceptible to high-altitude symptoms than others [11]. However, it has been proposed that symptoms could be mainly driven by an inefficient cardiopulmonary response, including an excessive elevation in PVR and RV overload, and the exacerbation of shunts, such as patent foramen ovale (PFO) or transpulmonary shunts, which can lead to more severe hypoxemia [11,12].

Hypoxic simulation testing (HSTs) simulates changes in physiology that occur at altitude by administering a mixture of gases with a lower O_2_ content to simulate different elevations [13]. These tests allow physicians to screen for significant hypoxia and potential signs and symptoms. The concomitant use of transthoracic echocardiography (TTE) during HSTs can detect the rise in RV pressures, changes in RV hemodynamics, and presence of shunts to help reveal underlying causes of symptoms. However, available studies that evaluated the effects of acute high-altitude simulation on RV hemodynamics produced conflicting results, and no consistent recommendations for the assessment of the cardiopulmonary response are available for HSTs [5]. Recognizing the physiological responses of the RV to acute hypoxia could be significant for gaining a better understanding of the mechanisms underlying signs and symptoms at high altitude.

The aim of this study was to use TTE during HSTs to assess changes in RV hemodynamics and potential shunts in patients referred for high-altitude symptoms.

## 2. Methods

A retrospective observational study of adult (≥18 years old) patients with unexplained dyspnea at high altitude who were referred to the Aerospace Medicine program at a single institution (Mayo Clinic Arizona) between 1 August 2021 and 31 March 2023 was performed. All patient underwent clinical assessment including arterial blood gas analysis and acid base determination prior to hypoxic exposure. Patients with a known history of cardiopulmonary diseases that could explain the presence of high-altitude symptoms were excluded from the study. The Mayo Clinic Institutional Review Board (IRB) approved this study (Approval Code: 23-006541, Approval Date: 7 August 2023).

### 2.1. Hypoxic Simulation Testing—Transthoracic Echocardiography

Demographic characteristics of the patients were assessed before the initiation of the HSTs. During HSTs, reduced FiO_2_ was administered for 20 min by asking the patients to breathe a hypoxic gas mixture of oxygen balanced with nitrogen through a tight-fitting mask. Different FiO_2_ concentrations to simulate altitude levels specific to patients’ history were used (from 6500 to 14,000 ft). Heart rate, arterial blood pressure, O_2_ saturation, and endtidal carbon dioxide (ETCO_2_) were monitored during the tests and both baseline and peak hypoxia echocardiographic measurements were collected.

**Figure 1:**
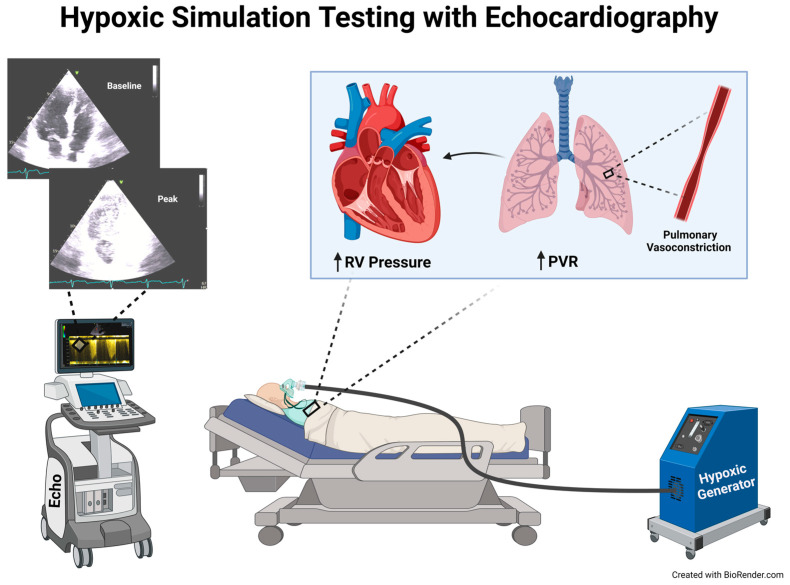
Illustration of Hypoxic Simulation Testing with the use of Transthoracic Echocardiography. During this test, decreased FiO_2_ was administered by asking the patients to breathe a hypoxic gas mixture. Vital signs were monitored, and echocardiography measurements were obtained during the tests, both at baseline and peak hypoxia. This physiological test led to hypoxia, pulmonary vasoconstriction, increase in pulmonary vascular resistance and right ventricle pressure in a susceptible population. Created with BioRender.com.

TTEs were performed using standard ultrasound scanners (Philips iE33; Phillips Medical Systems, Andover, MA, USA; GE Vivid E9, GE Healthcare, Milwaukee, WI, USA) with a 3.5 MHz transducer. All measurements were performed as per American Society of Echocardiography (ASE) guidelines [14]. Measurements were made using electronic calipers. For 2-dimensional images and for Doppler, an average of two measurements were used. All patients were in sinus rhythm and all measurements were performed by a single experienced cardiologist with an American Society of Echocardiography certification for special competency in echocardiography (RA). Patients were positioned in a supine position at 45 degrees. TTE images were obtained at baseline and during peak hypoxia to evaluate RV hemodynamics, including (1) RV systolic pressure, (2) RV systolic function by estimating the tricuspid annular plane systolic excursion (TAPSE), the maximum velocity achieved by the lateral tricuspid annulus during systole (S’ wave), and the RV free wall longitudinal strain, and (3) any possible shunt (Figure 1).

The RV systolic pressure was estimated utilizing the tricuspid regurgitation jet velocity and the evaluation of right atrial pressure based by the measurement of the inferior vena cava size and collapsibility. This measurement was only taken when a complete tricuspid regurgitation Doppler jet was evident. TAPSE was obtained by M-mode TTE with the cursor properly aligned along the direction of the tricuspid lateral annulus in the apical 4-chamber view to measure the displacement of the tricuspid ring in the longitudinal plane of the RV. S’ wave was obtained to reflect the peak lateral tricuspid annular systolic velocity by using Doppler tissue imaging, keeping the basal segment of the RV and the annulus aligned with the Doppler cursor. RV free wall longitudinal strain was measured for better estimating RV systolic function by calculating the percentage of systolic shortening of the RV free wall from base to apex using the RV-focused 4-chamber view. TomTec Software^®^ version 2.0 (TOMTEC Imaging Systems, Unterschleißheim, Germany) was used for offline evaluation of RV free wall longitudinal strain.

To detect any possible shunt, agitated saline tests were performed at baseline and maximum hypoxia according to ASE guidelines. The bolus injection of agitated saline contrast was composed of normal saline with room air. This mixture was then agitated back and forth between two sterile syringes right before the injection through a forearm vein. In the absence of shunts, no visible bubbles will cross the pulmonary circuit and will not be seen in the left cardiac chambers. A test was considered positive if visible bubbles appeared in the left chambers, indicating the presence of a shunt. If bubbles appeared immediately (within the first three beats after being noted in the right side of the heart), then the shunt was considered intracardiac. Otherwise, if bubbles arrived at the left-sided chambers after the third beat, then the shunt was considered more likely to be transpulmonary. If this test was negative under spontaneous conditions, Valsalva maneuver was performed [15]. In the cases in which Valsalva was indicated, patients were asked to force the expiration against a closed glottis. An effort was made to transiently increase the right atrial pressure by the release of the Valsalva maneuver right at the moment when the saline contrast bolus arrived in the right atrium. 

### 2.2. Statistical Analysis

Statistical comparison between baseline and peak hypoxia results was performed using Student’s paired t-test for continuous variables and McNemar test for categorical variables comparison. Statistical analyses were conducted using IBM SPSS Statistics, version 28.0 (IBM Corporation, Armonk, NY, USA). Data were presented as means with standard deviations (mean ± SD) for continuous variables and frequencies and percentages for categorical variables; *p*-values of <0.05 were considered statistically significant for all analyses.

## 3. Results

The study included 27 patients, with a mean age of 64.7 ± 14.4 years and 14 (51.9%) females. At the baseline TTE, normal mean values were noticed for left ventricle ejection fraction (LVEF) (62.7 ± 4.7%), TAPSE (20.4 ± 3.9 mm), S’ wave (12.7 ± 2.8 cm/s), RV free wall longitudinal strain (−23.8 ± 4.8%), and RV systolic pressure (27.7 ± 7.1 mmHg). No patient showed TTE signs compatible with increased left heart filling pressures and no significant cardiac valve diseases were detected. Furthermore, no significant abnormalities were seen in the rest of the baseline TTE parameters (Table 1).

Most of the patients (59.3%) received a mixture of gases to simulate an altitude of 8000 ft according to the altitude at which they referred symptoms. Regarding the comparison between baseline and peak hypoxia results, no significant differences in blood pressure and heart rate were detected, while significant reductions were seen in O_2_ saturation (96.1 ± 2.0 vs. 81.6 ± 9.2%, *p* < 0.001) and ETCO_2_ (28.9 ± 6.9 vs. 25.4 ± 6.1, *p* = 0.003) (Table 2). Notably, at peak hypoxia, there was a significant increase in RV systolic pressure (27.7 ± 7.1 vs. 36.6 ± 10.6 mmHg, *p* < 0.001), while RV systolic function declined as shown by a decrease in S’ wave (12.7 ± 2.8 vs. 11.3 ± 3.0 cm/s, *p* < 0.001), TAPSE (20.4 ± 3.9 vs. 19.1 ± 4.5 mm, *p* = 0.028), and RV free wall longitudinal strain (−23.8 ± 4.8 vs. −21.3 ± 5.4%, *p* < 0.001) (Table 2).

**Figure 2:**
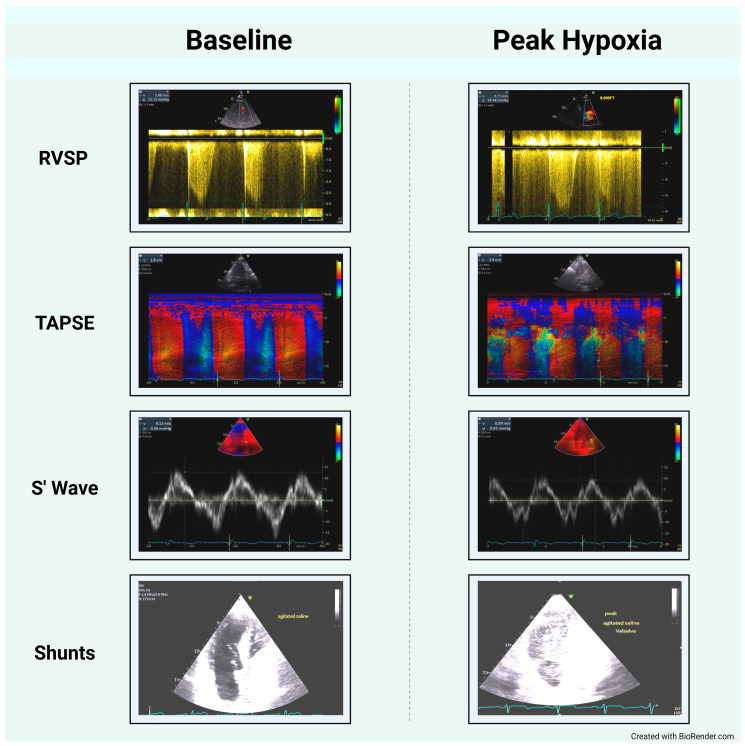
Echocardiography images illustrate the main differences between baseline and peak hypoxia measurements. Assessment of right ventricular systolic pressure, right ventricle systolic function (TAPSE, S’ wave), and the presence of shunts. TAPSE: Tricuspid Annular Plane Systolic Excursion. S’ wave: lateral tricuspid annulus peak systolic velocity.

Additionally, right-to-left shunting was identified in 19 (70.4%) patients. Of these, 17 (89.5%) were PFO and 2 (10.5%) intrapulmonary shunts. In four (21.1%) patients, the shunt was evident at baseline with no changes at peak hypoxia, while the severity of shunt increased at peak hypoxia in eight (42.1%) cases, and the shunt was only evident during hypoxia in seven (36.8%) patients (Figure 2).

## 4. Discussion

In this study, the use of TTE during HSTs demonstrated that patients with limiting symptoms at high altitudes had a significant and substantial increase in RV systolic pressure and a significant decrease in RV systolic function at high elevation simulation (peak hypoxia). Moreover, the combination of HSTs and TTE allowed both the observation of clinically occult shunts at baseline and the detection of exacerbation of PFOs that were mild or insignificant at baseline.

Prior investigations suggested that PFO may play a role in high-altitude symptoms. In 2006, Allemann et. al. demonstrated that PFO was 4 to 5 times more frequent in high-altitude pulmonary edema-susceptible mountaineers than in climbers resistant to that condition [12]. Moreover, authors demonstrated the contribution of PFO to an exaggerated arterial hypoxemia at high altitudes as the participants with large defects had more severe reductions in arterial oxygen saturation (65% vs. 77%, *p* = 0.02). An additional study by West et al. showed a higher prevalence of PFO in mountaineers with acute mountain sickness (AMS) compared to climbers without the condition. In that study, a multivariate model adjusted for age, gender, the use of AMS prophylaxis (medications which can alleviate symptoms of high altitude), and acclimatization (if participants spent more than two days at an altitude above 8000 ft) demonstrated that the presence of a PFO significantly increased the risk of AMS [11]. In contrast, a study by DiMarco et al. including 36 patients who were exposed to 7–10 h of normobaric hypoxia equivalent to 15,000 ft reported that hypoxic conditions were not associated with an increased incidence of high-altitude symptoms in patients with a PFO, and the presence of a PFO did not result in significant differences in pulmonary pressure [14]. Our findings are concordant with the first two studies, as we found shunts in 70% of our symptomatic population, a percentage that is substantially higher than what is observed in general population [15]. Additionally, most of the cases in our study had shunts that were only evident in hypoxia or that substantially increased their severity at peak hypoxic stress, thus suggesting a role for these shunts in generating clinical changes. Therefore, the absence of a PFO or a PFO with no apparent hemodynamic effects during a standard TTE should not lead to discounting of its potential role in the symptoms of patients complaining of high-altitude limitations. As a side note, the Valsalva maneuver was used as a sole provocation test to confirm or refute significant right-to-left shunt if the agitated saline test was negative under spontaneous conditions. There are potential limitations of using this approach, as the Valsalva maneuver depends on patient effort to induce the physiological temporary increase in right-sided pressures, opening to potentially false negative testing. Also, false negative agitated saline tests can occur if the interatrial septum is persistently displaced toward the right atrium during the injection in a way that the PFO remains closed with the septum. In cases of high clinical PFO suspicion in symptomatic patients, a repeat agitated saline injection should be conducted utilizing a blood-saline–air mixture, a more accurately performed Valsalva maneuver, and potentially using transesophageal echocardiography instead of TTE. 

Acute hypoxia exposure is consistently associated with an elevation of pulmonary arterial pressure resulting in an increased load on the RV [5,16]. However, the RV responses to this increased afterload are not completely characterized. Although increased RV afterload is considered the main factor that predisposes to RV dysfunction in this scenario, other physiological mechanisms have been proposed. Prior publications proposed direct effects of hypoxia on the myocardial contractility through the activation of various pathways (oxidative stress, protein kinase activation, and inflammatory processes) [17]. These alterations could lead to RV dysfunction. However, those mechanisms have been mainly evaluated after chronic exposure to hypoxia. Additionally, a preclinical study utilized cardiac myocytes for the evaluation of acute exposure to severe hypoxia [18]. This investigation demonstrated that there was a transient reduction in myocyte contractility along with a decrease in the amplitude of the intracellular Ca^2+^ in hypoxic cells. Furthermore, lactate production stayed transitory elevated after the return to normal O_2_ pressure. Another animal study investigated the impact of systemic hypoxia on myocardial contractility through the administration of a gas mixture with 10% O_2_ during a 15-min session [19]. After this period, a novel intervention was tested; one group of lambs received an O_2_ delivery biotherapeutic and the other group did not receive it. First, the acute exposure to hypoxia generated a significant rise in heart rate, pulmonary blood flow, and PVR. Moreover, after 1 h, non-treated lambs showed a substantial impairment in biventricular contractile function, while the treated group demonstrated an improvement in myocardial oxygenation without a negative impact in systemic resistances or PVR. In animals that received the O_2_ delivery biotherapeutic, both RV and left ventricle contractile function remained stable in comparison to pre-hypoxic levels. 

Regarding clinical studies evaluating RV function after an exposure to hypoxia, a systematic review including 37 studies (12 using HSTs and 25 conducted in real high-altitude conditions) concluded that, despite consistent increases in pulmonary pressure across the included studies, the effects of acute hypoxia on RV hemodynamics are controversial and inconclusive [5]. The lack of consistent findings in regard to RV function could be highly influenced by substantial variations in study designs, highly different approaches to RV function assessment, and restricted statistical power due to insufficient sample sizes [5,16,20]. Our study, however, was able to demonstrate not only the increase in RV pressure but also a significant decrease in RV systolic function using widely available RV echocardiography parameters, thus supporting the negative impact of the acute increase in PVR and pressure overload on RV hemodynamics. Additionally, we measured the RV free wall longitudinal strain to overcome the potential limitations of traditional RV function parameters. In comparison to TAPSE and S’ wave, RV longitudinal strain is angle-independent, less load-dependent, estimates the regional myocardial deformation, and is capable of detecting subclinical RV dysfunction even when traditional methods for RV systolic function are still in the normal range. 

Considering the lack of large prospective studies and the controversial evidence produced so far, the clinical use of HSTs remains currently limited. Moreover, previous studies reported significant differences in hypoxemia, hypocapnia, and pulmonary pressure results during exposure to altitude and HST, thus questioning its use to accurately predict physiological changes at altitude [21]. Therefore, the clinical use of HST is currently limited in its ability to predict inflight O_2_ saturation in chronic obstructive pulmonary disease (COPD) patients with borderline O_2_ saturation at sea level and to recommend supplemental inflight O_2_ [22,23]. HST ability to predict adverse events at high altitudes has not been thoroughly evaluated [24], no consistent results have been reported, and no uniform recommendations exist regarding the use of TTE during these tests. 

Based on our results, we propose an approach for the concomitant use of HST and TTE to better understand the development of adverse symptoms during altitude exposure. First, the gas mixture used during HST should be adapted to each patient’s exposure history to try to simulate the altitude and implicit hypoxic burden at which symptoms developed. TTE measurements should be obtained at baseline and during peak hypoxia focusing on RV pressure measurements, easy-to-reproduce RV systolic function parameters, and the presence and severity of any potential shunts. The use of provocation maneuvers, such as Valsalva, is critical to detect shunts that are unobserved under spontaneous conditions. 

The limitations of our study are related to its observational nature, the absence of a control group, and the lack of invasive measurements (right heart catheterization) to better understand hemodynamic variations and transesophageal echocardiography (TEE) to improve the evaluation of the shunts. Additionally, in this proof-of-concept study, TTEs were focused on RV function and pressure; therefore, limited data were available to evaluate the ratio between pulmonary and systemic flow. As an observational retrospective study, establishing causal relationships is not possible, and the lack of a control group restricts our ability to control for confounding variables. Our study included exclusively a cohort exhibiting known high-altitude symptoms, thus limiting the generalizability of our findings to this specific population. One additional constraint is the reliance on a relatively small sample size, which, although sufficient for detecting statistical differences between baseline and peak hypoxia results, renders it inadequate for conducting granular comparisons based on demographic variables such as age and sex. Considering the above mentioned, our results serve as a foundation for generating hypotheses rather than definitive conclusions. To address these limitations and enhance the validity of our findings, additional studies with larger and more diverse participant samples are imperative to explore the relationship between PFO, RV hemodynamics, RV pressure, and adverse symptoms at high altitudes. Future research efforts should incorporate control groups (patients with no altitude-related symptoms), include more extensive demographic stratification, incorporate invasive measurements and TEE, and explore potential confounding variables to provide a more comprehensive understanding of the complex interplay between these factors.

In terms of future perspectives and implications, the application of HST in this scenario could potentially yield therapeutic benefits. The effective management of exacerbated pulmonary pressure increases and the resolution of shunts within this susceptible population remain a topic of debate. Exploring these strategies in more detail is essential and should be a focal point in upcoming prospective studies. One possibility for such exploration could involve conducting a randomized trial that compares the efficacy of PFO closure to standard therapy to comprehensively determine whether PFO closure holds the potential to alleviate the symptoms associated with high-altitude exposure in susceptible populations.

## 5. Conclusions

Echocardiography during HST in this susceptible population provided valuable information by revealing the presence of sustained shunts and confirming the increase in right-sided pressure and the decline in right ventricle hemodynamics at peak hypoxia. These findings contribute to better explaining the development of dyspnea and worsening oxygenation at high altitude and may be of value in the investigation of alterations of cardiac function due to altered pulmonary pressures leading to shunting, and they may be of particular interest in the domain of congenital heart disease. Further studies are necessary to establish the optimal clinical role of this physiologic method and to explore potential therapeutic interventions in symptomatic patients. 

## Figures and Tables

**Table 1:** Demographic and baseline echocardiographic characteristics of the included population.

*n* = 27	
Age, years	64.7 ± 14.4
Sex, n (%)	
Female	14 (51.9%)
Male	13 (48.1%)
Height, m	1.72 ± 0.09
Weight, Kg	88.9 ± 19.7
BMI, Kg/m^2^	30.2 ± 7.3
BSA, m^2^	2.0 ± 0.2
TTE baseline parameters	
LVIVS, mm	9.6 ± 1.5
LVPW, mm	9.4 ± 1.0
LVEDD, mm	48.1 ± 5.6
LVEDV, mL	89.5 ± 21.9
LVESV, mL	33.9 ± 12.1
Ejection Fraction, %	62.7 ± 4.7
E wave	0.6 ± 0.2
A wave	0.6 ± 0.2
E/A ratio	1.1 ± 0.5
E/e’ medial	9.2 ± 5.3
E/e’ lateral	7.0 ± 4.5
Diastolic function (grading)	
Normal	16 (59.3%)
Grade 1	11 (40.7%)
Severe valvular heart diseases	0 (0.0%)
RV basal diameter, mm	35.9 ± 4.6
RV mid diameter, mm	30.8 ± 4.7
TAPSE, mm	20.4 ± 3.9
S’ wave, cm/s	12.7 ± 2.8
RV free wall strain, %	−23.8 ± 4.8
RVSP, mmHg	27.7 ± 7.1
HSTs Target altitude	
Target Altitude, ft (mean)	8766.7 ± 1794.2
Target Altitude, n (%)	
6500 ft	1 (3.7%)
7300 ft	1 (3.7%)
7500 ft	1 (3.7%)
8000 ft	16 (59.3%)
8200 ft	2 (7.4%)
10,000 ft	1 (3.7%)
11,500 ft	2 (7.4%)
12,000 ft	2 (7.4%)
14,000 ft	1 (3.7%)

BMI: Body Mass Index, BSA: Body Surface Area, TTE: Trans-Thoracic Echocardiography, LVIVS: Left Ventricular Inter-Ventricular Septum, LVPW: Left Ventricular Posterior Wall, LVEDD: Left Ventricular End Diastolic Dimension, LVEDV: Left Ventricular End Diastolic Volume, LVESV: Left Ventricular End Systolic Volume, TAPSE: Tricuspid Annular Plane Systolic Excursion, S’ wave: Systolic Velocity, RVSP: Right Ventricular Systolic Pressure, HST: Hypoxic Simulation Test.

**Table 2:** Comparison between baseline and peak hypoxia outcomes.

Parameter	Baseline	Peak Hypoxia	*p* Value (95% CI)
SBP, mmHg	129.2 ± 14.7	128.9 ± 14.1	0.886
DBP, mmHg	78.8 ± 9.0	77.5 ± 8.7	0.487
HR, bpm	67.4 ± 11.2	69.9 ± 12.5	0.148
O_2_, %	96.1 ± 2.0	81.6 ± 9.2	<0.001 (95% CI 10.9–18.2)
ETCO_2_, mmHg	28.9 ± 6.9	25.4 ± 6.1	0.003 (95% CI 1.3–5.6)
RVSP, mmHg	27.7 ± 7.1	36.6 ± 10.6	<0.001 (95% CI 6.0–11.8)
TAPSE, mm	20.4 ± 3.9	19.1 ± 4.5	0.028 (95% CI 0.2–2.4)
S’, cm/s	12.7 ± 2.8	11.3 ± 3.0	<0.001 (95% CI 0.6–2.2)
RV free wall strain, %	−23.8 ± 4.8	−21.3 ± 5.4	<0.001 (95% CI 1.0–3.9)

SBP: Systolic Blood Pressure, DBP: Diastolic Blood Pressure, HR: Heart Rate, O_2_: Oxygen, ETCO2: End-tidal Carbon Dioxide, RVSP: Right Ventricular Systolic Pressure, TAPSE: Tricuspid Annular Plane Systolic Excursion, S’ wave: Systolic Velocity.

## Data Availability

The data presented in this study are available on request from the corresponding author. The data are not publicly available due to privacy restrictions.
